# Improved Planning Abilities in Binge Eating

**DOI:** 10.1371/journal.pone.0105657

**Published:** 2014-08-22

**Authors:** Rémi Neveu, Dorine Neveu, Franck Barsumian, Elsa Fouragnan, Edouard Carrier, Massimo Lai, Jocelyne Sultan, Alain Nicolas, Giorgio Coricelli

**Affiliations:** 1 CNRS, UMR5292, INSERM, U1028, Université Lyon 1, Université de Lyon, Neuroscience Research Center, Team Olfaction: from coding to memory, Lyon, F-69366, France; 2 Praxis, Ville-la-Grand, France; 3 Université Montpellier 1, Montpellier, France; 4 INSERM U 1058, Montpellier, France; 5 CHU Montpellier, Département d'information médicale, Montpellier, France; 6 University of Trento, Trento, Italy; 7 Clinique Saint Vincent de Paul, Lyon, France; 8 Clinique Lyon-Lumière, Meyzieu, France; 9 Clinique Stella, Vérargues, France; 10 Hôpital du Vinatier, Bron, France; 11 University of Southern California, Los Angeles, California, United States of America; Inserm, France

## Abstract

**Objective:**

The role of planning in binge eating episodes is unknown. We investigated the characteristics of planning associated with food cues in binging patients. We studied planning based on backward reasoning, reasoning that determines a sequence of actions back to front from the final outcome.

**Method:**

A cross-sectional study was conducted with 20 healthy participants, 20 bulimia nervosa (BN), 22 restrictive (ANR) and 23 binging anorexia nervosa (ANB), without any concomitant impulsive disorder. In neutral/relaxing, binge food and stressful conditions, backward reasoning was assessed with the Race game, promotion of delayed large rewards with an intertemporal discounting task, attention with the Simon task, and repeating a dominant behavior with the Go/No-go task.

**Results:**

BN and to a lower extent ANB patients succeeded more at the Race game in food than in neutral condition. This difference discriminated binging from non-binging participants. Backward reasoning in the food condition was associated with lower approach behavior toward food in BN patients, and higher food avoidance in ANB patients. Enhanced backward reasoning in the food condition related to preferences for delayed large rewards in BN patients. In BN and ANB patients the enhanced success rate at the Race game in the food condition was associated with higher attention paid to binge food.

**Conclusion:**

These findings introduce a novel process underlying binges: planning based on backward reasoning is associated with binges. It likely aims to reduce craving for binge foods and extend binge refractory period in BN patients, and avoid binging in ANB patients. Shifts between these goals might explain shifts between eating disorder subtypes.

## Introduction

Binge eating episodes are periods of rash overeating that are commonly viewed as a failure of the strict control over food intake exerted by patients with bulimia nervosa (BN), anorexia nervosa binging subtype (ANB), or eating disorder not otherwise specified (EDNOS) [1], [2]. Patients' behaviors during binges question the common interpretation of loss of control during binges: patients can break off the binge if they are disturbed by an external event and resume binging thereafter [3], which suggests that they can inhibit their behavior; patients eat foods that they usually restrict outside binges [2]–[4], which suggests that binge foods are not chosen at random; patients can refrain from binging if environmental conditions, such as the availability to purge afterwards, are not met [3]; and some patients plan in advance their next binge [3]. Moreover, as food is a major personal concern in patients with an eating disorder that directs patients' attention [1], [5], food related behaviors may recruit planning skills [4], [6], [7]. Accordingly, selecting and collecting foods for a binge and setting the appropriate environment for their ingestion require planning [6].

Cognitive planning recruited during the intense binge craving period that occurs just before binge food ingestion [8], [9] may rely on one of the two opposite strategies: patients are to ingest binge foods (i.e. approach behavior toward binge foods) to relieve the stress related to craving [3], [10], and facilitate binge food restriction thereafter [11]; or patients are to avoid binge food cues to limit craving.

Binge food intake characteristics and sensitivity to outcomes depend on the subtype of eating disorder and might result from these strategies. ANB patients eat much less foods during binges than BN patients [3]; BN patients continue eating even after being satiated [12]; the nature and the intensity of craving differ between ANB and BN patients [9], [13]; ANB patients are sensitive to punishment only while BN patients are sensitive to reward and punishment [14]. These discrepancies suggest that the goal of the planning that occurs just before binge food ingestion may depend on the subtype of eating disorders.

To date, studies have failed to show any consistent association between planning and binges [15], [16], possibly because these studies used self-administered questionnaires that capture traits rather than instantaneous behaviors [17], and neuropsychological tasks that were performed in neutral settings only [18]–[21].

We investigated the characteristics of planning associated with food cues in BN and ANB patients. We focused on planning based on backward reasoning - reasoning that determines a sequence of actions back to front from the final goal [22]. We also investigated the association between backward reasoning and the following other cognitive control skills: discounting of future rewards, repeating and inhibiting an automated behavior, and attention paid to binge foods. We hypothesized that BN and ANB patients exhibit enhanced backward reasoning when exposed to binge food cues compared to neutral relaxing cues while restrictive anorexia nervosa (ANR) patients and healthy participants would not. We also hypothesized that these enhanced planning skills are associated with the promotion of long-term rewards and reduction of approach behavior toward binge food in BN patients (i.e. relief of binge food craving) and with binge food avoidance in ANB patients.

## Methods

### Study design and population

We conducted a cross-sectional study. We recruited four groups of women, aged 18–35 years, with a body mass index (BMI) <25 kg/m^2^: individuals with a current diagnosis of binging anorexia nervosa (ANB, N = 23), restrictive anorexia nervosa (ANR, N = 22, control group for ANB to account for anorexia nervosa), or bulimia nervosa with or without purging behaviors (BN, N = 20) (DSM-IV R criteria), or individuals free of any eating disorder (controls, N = 20, control group for BN). Only data of participants who fully completed the neuropsychological assessments were analyzed (i.e. 16 ANR, 19 ANB, 18 BN and 18 controls). All patients were stable for antidepressant, anxiolytic and neuroleptic medication for more than one week [23], [24] to avoid interaction with neuropsychological performances. Exclusion criteria included: any addiction, histrionic personality disorder, psychotic disorder, dementia or mental retardation, and the following impulsive disorders: antisocial personality disorder, attention deficit and hyperactivity disorder, borderline personality disorder, intermittent explosive disorder (DSM-IV R criteria assessed by a trained psychiatrist with a structured clinical interview [25]). Controls were recruited through e-mail advertisements and patients through inpatient units specialized in the treatment of eating disorders (Lyon, Meyzieu, Ville-la-Grand, Vérargues; France). All participants provided written informed consent before inclusion. The study was approved by an independent ethical committee, Comité de Protection des Personnes Sud Est IV.

### Assessment

Participants were randomly allocated to morning or afternoon assessment to account for circadian variations in binge occurrence [26] with stratification by site and diagnosis.

#### Tasks

**Figure 1:**
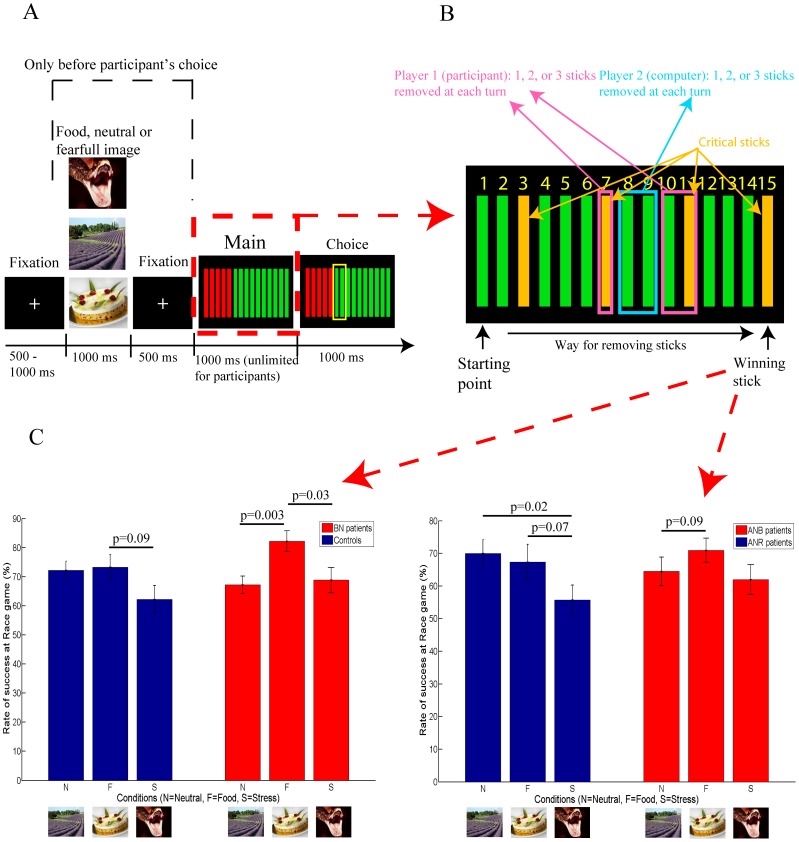
Race Game trial design (A), critical steps that allow the first player to systematically win if critical sticks are removed (B), and mean success rate in BN and ANB patients (red), in controls and ANR patients (blue) in neutral/relaxing, food and stress/fearful conditions (C). Vertical bars are standard error of the mean (C).

Participants underwent a computerized version of the Race game [22] for two players (Figure 1A). This game assesses backward reasoning abilities [22] and focuses attention to the final outcome. Each player, at their turn, can remove one to three sticks simultaneously. The winner removes the last stick 15. The backward reasoning goes as follows: focusing on the winning stick 15, participants realize that if they remove the 11^th^ stick, they win whatever the computer removes thereafter (Figure 1A). To make sure to remove the 11^th^ stick, participants must remove stick 7, and with the same reasoning stick 3. The critical sticks are 3, 7 and 11. All participants were told that they were going to play 20 games against the computer. The computer applied the winning strategy at critical stick 11 (the computer endeavored to remove stick 11 and did not endeavor to remove critical sticks 3 or 7, easy level for the participant), 7 (the computer endeavored to remove sticks 7 and 11, intermediate level) or 3 (the computer endeavored to remove sticks 3, 7 and 11, difficult level). The computer was allowed to fail the winning strategy in 5% of cases to mimic a “human's” lack of attention. Participants always started.

In addition, participants performed an intertemporal discounting task [27] that assessed the preferences for larger and delayed rewards over sooner and smaller rewards. Participants performed 75 trials with two options: i) immediate with a payoff ranging randomly from 0€ to 10€, ii) delayed with a fixed 10€ payoff and a random delay in the range 1–365 days. Participants also performed the Simon task [28] (*i.e.* the non-verbal Stroop task) that assesses the resistance to interference, and the Go/No-go task [29] that assesses the ability to repeat (go trials) and inhibit (no-go trials) an automated behavior (supplementary methods in file S1).

Participants received a 60€ fixed payment and a variable payment based on one choice randomly selected in the intertemporal task [30]. All tasks were computerized using Presentation software (NeuroBehavioralSystems, release 14.2, Albany, CA, USA).

#### Preconditioning

Each task was assessed in the framework of three preconditioning situations: food (25% of games or trials), stressful (25% of games or trials) or neutral/relaxing (50% of games or trials). Image cues were displayed for one second followed by a 500 ms fixation cross (Figure 1B) before each trial or participants' turn in the Race game. Images were neutral/relaxing or fearful (from the International Affective Picture System) or binge foods designed to induce craving for binging [8]. Food images were selected from the lists of binge foods sorted by increasing craving induction for the binge established by two BN patients who did not perform the tasks. One patient was more attracted by salty foods, the other one by sweet foods.

Participants were told that these images were used to set a specific context (neutral/relaxing, food and stressful) and were not related to the task itself to prevent any anticipation about the upcoming stimuli (monetary options at the intertemporal discounting task, letter at the Go/No-go task, type or arrow at the Simon task) or strategy of the computer at the Race game.

In the Race game, games were randomized across conditions. Levels of artificial intelligence were counterbalanced across neutral conditions and stress inducing conditions (i.e. food and stressful). In the three other tasks, neutral/relaxing, fearful and binge-food priming images were displayed at random before each trial. At the end of the neuropsychological assessment the anxiety aroused by every image was assessed with a continuous digital scale ranging from 0 to 100, where 0 referred to an absence of anxiety and 100 to a life threatening situation.

#### Data collection

Weight was measured with a 0.1 kg precision and height with a 1 mm precision. Socio-demographic characteristics, mathematical knowledge, educational level and father socio-economic status [31] were collected with a self-administered questionnaire.

### Statistical considerations

#### Race game

For each participant and each game, we measured three parameters: cumulated reaction time between two consecutive critical sticks (sticks 1 and 3 included, 4 and 7 included, 8 and 11 included, 12 and 15 included), backward reasoning (number of consecutive critical sticks optimally removed, i.e. back to front order, sticks 15, 11, 7 and 3: losing a game gets 0, winning a game by removing stick 15 only gets 1, by removing sticks 11 and 15 gets 2, and so on), and success (yes/no). For each condition and each participant we averaged reaction times and backward reasoning over games and computed the success rate, defined as the ratio of the number of successes to the total number of games in that condition.

#### Intertemporal discounting task

For each condition and participant, the discount process of delayed options (k parameter) was estimated using a hyperbolic discounting function [27] fitted over participants' choices using a probabilistic approach (supplementary methods in file S1). An increase in k refers to an increase of choices of the immediate option versus the delayed option.

#### Other neuropsychological tasks

For each participant, we estimated reaction time and coefficient of variation (ratio of standard deviation to mean, CV) for correct Go trials, rate of errors for No-go trials, and reaction time for correct congruent and incongruent trials in the Simon task. Interference effect was the difference in reaction times between correct incongruent and correct congruent trials in the Simon task.

#### Statistical analysis

“Food-specific” parameters were defined as the difference between the parameter estimates in food versus neutral conditions.

Quantitative variables with non gaussian distribution were compared within groups using the paired Wilcoxon test and between groups with the Mann-Whitney or Kruskal-Wallis tests. Success rate and backward reasoning in the Race game, k in the intertemporal discounting task, interference effect in the Simon task and anxiety ratings in neutral, food and stressful conditions were not normally distributed in any of the four groups of participants (p<0.00007, Kolmogorov-Smirnoff test).

The performance of planning enhancement between food and neutral/relaxing conditions to discriminate BN versus controls, ANB versus ANR or binging patients (BN and ANB) versus non binging participants (ANR and controls) was estimated with receiver operating characteristic (ROC) curves established for “food specific” success rate at the Race game.

**Figure 2:**
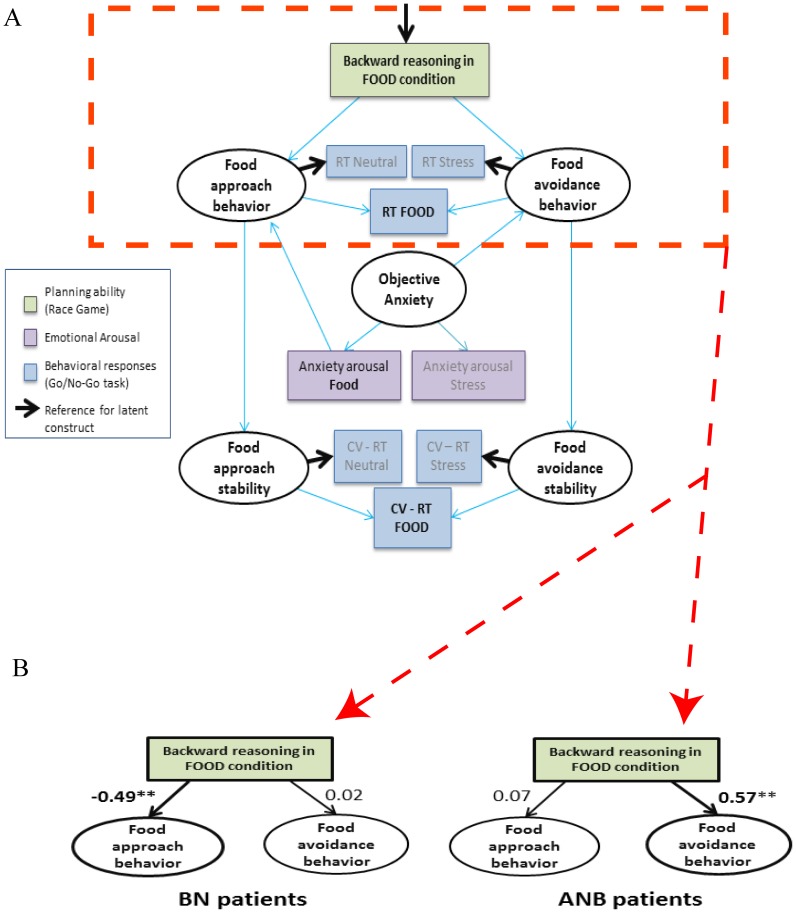
Model used to investigate the association between backward reasoning in the Race Game in the food condition and approach or avoidance behaviors, under the influence of anxiety (Figure A). Food approach behavior was determined by the shared variance of the reaction time (RT) at correct Go trials between food and neutral/relaxing conditions with neutral/relaxing data as a reference. Food avoidance behavior was determined in a similar manner, using the stressful and food conditions. Similar reasoning was carried out for “food specific” anxiety arousal and coefficient of variation of reaction time (CV-RT). Bold arrows represent reference loadings for latent variables (ellipses) and have a factor loading set to 1. Standardized coefficients are reported for Bulimia Nervosa (BN) and Anorexia Nervosa Binging subtype (ANB) patients (Figure B and supplementary table ST4 in file S2). **: p<0.05.

To investigate whether BN and ANB patients had distinct goals of planning in the food condition, we compared the influence of backward reasoning (Race game) on approach and avoidance behaviors between BN and ANB groups, using Structural Equation Modeling (SEM, lavaan package in R software, SEM 1 for BN patients and SEM 2 for ANB patients). SEMs permit some degree of inference regarding directional relationships between variables. Because binge foods are highly palatable [32], [33] and highly avoided by patients [1], reaction time in the food condition in the Go/No-go task results from both approach and avoidance behaviors. To distinguish these two behaviors in the food condition, we built the food approach behavior as the shared inter-subject variance of reaction time for correct go trials between food and neutral/relaxing conditions (Figure 2A). Similarly, food avoidance behavior was built using the stressful instead of neutral condition. To make sure that latent constructs referred to approach or avoidance, loading of the appropriate observed parameter was set to 1 (bold arrows on Figure 2A). Because binges often include foods with high palatability [32], [33] that are associated with enhanced emotional arousal [34], we also included anxiety in the food condition as a regressor of approach behavior. We also included behavioral stabilities (referred as approach and avoidance stabilities) for convergence purposes (Figure 2A).

We estimated SEM parameters with a weighted least square method and assessed model fitting with chi square and RMSEA criteria [35]. Backward reasoning rather than success rate at the Race game was used for convergence purpose.

Because ANR patients are characterized by an exclusive avoidance of food intake [1], we compared the pattern of associations between the success rate in the Race game and behaviors (approach and avoidance) in ANB with those patterns of associations in BN and also with those in ANR to check the reliability of SEM results. Associations are detailed in supplementary methods in file S1.

Because planning abilities are associated with the choice of delayed rewards [36], we investigated whether improvement in backward reasoning in food compared to neutral conditions was associated with a lower discount parameter k in the intertemporal discounting task, with a Pearson correlation coefficient. One BN and one ANB with k>3SD were excluded from this latter analysis.

We investigated whether a higher “food specific” success rate in the Race game was associated with a higher attention paid to food. Attention to food was assessed by the “food specific” interference effect in the Simon task. A linear model (using matlab anovan function) included the “food specific” interference effect as a function of the “food specific” success rate in the Race game, group (ANB/BN) and their interaction.

All tests were two tailed except for ROC curves. P-values were corrected for multiple testing using the Benjamini-Hochberg correction [37]. Analyses were carried out with R software (release 2.14.1) and Matlab (release 2011a, Mathworks Inc).

## Results

The four groups had similar inhibitory controls (error rate at no-go trials), interference levels (difference of reaction times between correct incongruent trials and correct congruent trials in the Simon task), educational levels, university curricula, mathematical knowledge and father socio professional status (table 1, table 2 and supplementary table ST1 in file S2). On average, ANR patients were younger than the other participants.

**Table 1:** Socio demographic characteristics for the four groups.

Socio-demographic characteristics	n	Bulimia nervosa	n	Controls	n	Anorexia nervosa binging subtype	n	Anorexia nervosa restrictive subtype	p-value
Age, year	18	24.2 (5.78)	18	24.3 (3.21)	19	25.6 (4.92)	16	21.7 (5.04)	0.046
Educational level, years	14	12.8 (2.23)	18	14.2 (2.43)	14	14.1 (2.96)	16	13.3 (1.59)	0.2
Paternal socio-professional status, n (%)	15		18		15		13		0.5
INSEE 1 or 2		1 (6.67)		2 (11.1)		2 (13.3)		2 (15.4)	
INSEE 3		5 (33.3)		6 (33.3)		6 (40)		7 (53.8)	
INSEE 4, 5 or 6		6 (40)		8 (44.4)		3 (20)		4 (30.8)	
INSEE 7 or 8		3 (20)		2 (11.1)		4 (26.7)		0 (0)	
Knowledge about fuzzy set theory and probabilities, n (%)	18	0 (0)	18	2 (11.1)	19	1 (5.26)	16	1 (6.25)	0.27
Computer science, mathematics, physics, chemistry or business program at university, n (%)	18	1 (5.56)	18	1 (5.56)	19	1 (5.26)	16	1 (6.25)	1

Mean (standard deviation) are reported for quantitative parameters.

INSEE #: socio-professional category according to the “Institut National de la Statistique et des Etudes Economiques”: 1,2: Farmer, craftsman, shopkeeper and large retailer, chairman and managing director; 3:senior executive, manager; 4,5,6: intermediate jobs, employees and workers; 7,8: retired, without any job.

**Table 2:** Behavioral characteristics in neutral condition for the four groups.

Behavioral characteristics	Bulimia nervosa (n = 18)	Controls (n = 18)	p-value	Anorexia nervosa binging subtype (n = 19)	Anorexia nervosa restrictive subtype (n = 16)	p-value
Error rate at no-go trials, %	2.2 (4.6)	4.8 (10.2)	0.48	2.8 (5.6)	2.1 (4.0)	0.88
Rate of good responses at go trials, %	100 (0)	99.8 (0.5)	0.34	100 (0)	99.7 (0.8)	0.13
Error effect* at Simon task, %	−2.4 (4.4)	−4.0 (5.5)	0.42	−5.9 (9.2)	−9.1 (21)	0.57
Interference effect** at Simon task, ms	63 (77)	60 (60)	0.44	69 (73)	44 (46)	0.44

Mean (standard deviation) are reported for quantitative parameters.

*Error effect: difference of error rates between incongruent and congruent trials **Interference effect: difference of reaction times between incongruent and congruent trials.

### Anxiety arousal

Fearful as well as food images aroused significantly higher anxiety level than neutral/relaxing images in ANR, ANB and BN patients. In controls, fearful images aroused significantly higher anxiety level than neutral/relaxing ones, and neutral/relaxing and food images aroused similar anxiety levels (Supplementary figure SF1 in file S2). Neutral/relaxing images aroused higher anxiety level in ANR, ANB and BN patients than in controls (supplementary figure SF1 in file S2, p = 0.0008, p = 0.05, p = 0.0008 respectively).

### Planning ability and its goal (Race game and Go/No Go task)

**Figure 3:**
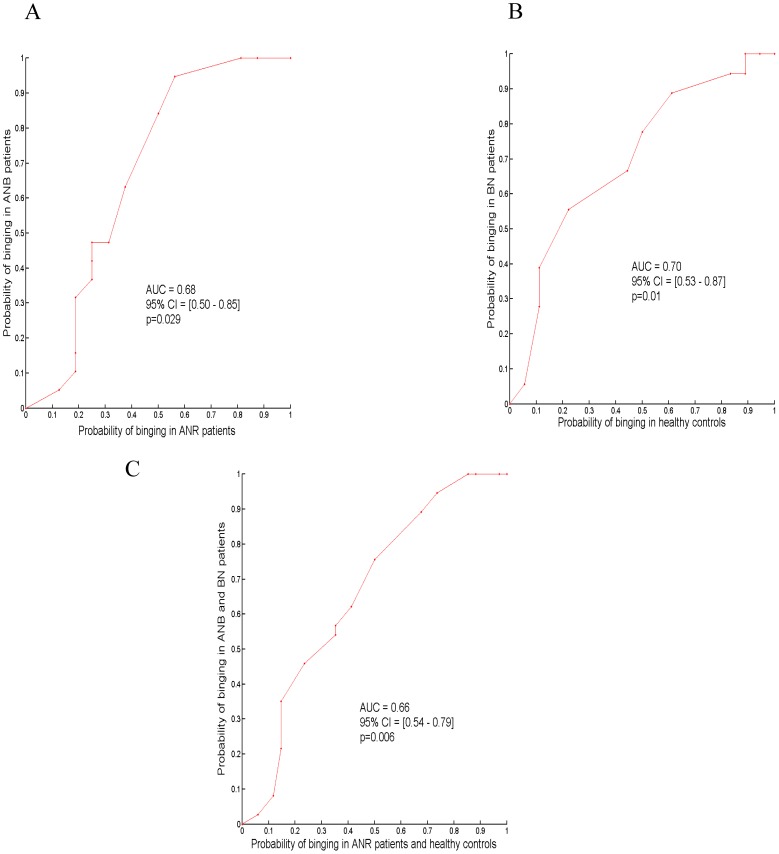
Receiver Operating Characteristic Curves (Figures A, B and C) for the difference in rate of success in the Race game between the food and neutral conditions for the detection of binging status in patients with anorexia nervosa restrictive subtype (ANR) and binging subtype (ANB) (Figure A), in patients with bulimia nervosa (BN) and healthy controls (Figure B) and in the four aforementioned groups (Figure C). Statistic tests were one tail. Abbreviations: AUC, area under curve. CI, confidence interval.

Figure 1 C shows success rates in the Race game in each group and each condition. The “food specific” success rate in the Race game was higher in binging patients than in their respective controls (mean (SD): 15% (15.4%) in BN patients versus 1.1% (21.7%) in BN healthy controls, p = 0.04; 6.5% (20.1%) in ANB patients versus −2.6% (30.7%) in ANR patients p = 0.08; supplementary table ST2 in file S2). This “food-specific” improvement in success rate in the Race game discriminated binging from non-binging participants (Figure 3). “Food-specific” improvement in success rate in the Race game was not statistically associated with younger age in AN patients (ANB and ANR grouped) (r = −0.13, p = 0.44).

In all groups, participants developed a strategy in the Race game: the last four sticks (12 to 15) were removed more quickly than the four previous sticks (8 to 11), which were removed as quickly or more slowly than sticks 4 to 7, whatever the condition (supplementary figure SF2 in file S2). Backward reasoning was significantly associated with the rate of success in the four groups, whatever the condition (supplementary table ST3 in file S2).

Results from the Structural Equation Model show that backward reasoning in the food condition was inversely associated with approach behavior in BN patients (SEM 1, path between food backward reasoning and food approach behavior: −0.49, p = 0.03, figure 2B and supplementary table ST4 in file S2), while it was associated with avoidance behavior in ANB patients (SEM 2, path between food backward reasoning and food avoidance behavior: 0.57, p = 0.04, figure 2B and supplementary table ST4 in file S2).

### Performances in the intertemporal discounting task and their relationship with planning abilities

BN and ANB patients discounted future reward less in food than in neutral/relaxing condition while ANR patients did not: the discount rate k parameter was lower in food than in neutral conditions (mean difference (SD): −1.68×10^−3^ (4.83×10^−3^) days^−1^, p = 0.005 for BN patients, and −1.05×10^−3^ (1.63×10^−3^) days^−1^, p = 0.01 for ANB patients, −0.1×10^−2^ (8*10^−2^), p = 0.84 for ANR patients). The “food specific” k decreased with increasing “food specific” backward reasoning in BN patients only (BN: r = −0.48, p = 0.05; ANB: r = 0.33, p = 0.18; ANR: r = 0.28, p = 0.33; Controls: r = 0.2, p = 0.43).

### Orientation of attention (Simon Task)

Attention tended to be captured more by food than by neutral/relaxing images in binging patients (BN and ANB grouped, mean (SD): 90 (70) vs. 66 (74) ms, respectively, p = 0.08). “Food specific” attention tended to be higher in binging patients than in non-binging participants (controls and ANR grouped): mean (SD): 24 (74) vs. −9 (61) ms, respectively, p = 0.07. “Food specific” success rate in the Race game was associated with “food specific” attention in binging patients (Beta = 2.93×10^2^ ms/%, p = 0.02).

## Discussion

This study shows that planning ability based on backward reasoning assessed in the Race Game is enhanced in the binge food condition in comparison with neutral/relaxing or generically stressful conditions in binging patients, BN and to a lower extent in ANB. This effect was not found in non-binging participants, ANR patients and healthy participants. In BN patients, this enhancement was associated with a reduction in food approach behavior, measured by the go trials responses at the Go/No-go task, and the promotion of larger delayed rewards assessed in the intertemporal discounting task. In contrast, in ANB patients the enhancement of backward planning was associated with an enhanced avoidance of binge foods.

The enhanced backward reasoning in binge condition may aim to organize the binge to extend binge refractory period afterwards. In line with this, we showed that, in BN patients, the enhanced backward reasoning in the binge food condition is associated with more frequent choices of a delayed large reward. Moreover, BN patients continue eating even after being satiated, while ANB do not [12], resulting in a larger food intake during binges than in ANB patients [3]. Also, BN patients resume binging once disturbance is over [3] and are sensitive to rewards while ANB patients are not [14]. These findings support the hypothesis that a “planned” goal oriented behavior underlies binge food ingestion in BN patients [12], [14]; the goal being craving relief and a longer binge refractory period after the binge.

In conclusion, planning ability was enhanced in the binge food condition through backward reasoning in binging patients contrary to ANR patients and healthy controls. This enhancement would aim to relieve craving and to extend the refractory period following the binge in BN patients; and it would aim to avoid the binge in ANB patients. These results have several implications.

First, these findings might explain shifts between ANR, ANB and BN profiles [1]. When strict dieting becomes less effective in terms of weight loss, ANR patients might increase their control over food intake in order to compensate, and this increase of control might be expressed through planning improvement. The present findings suggest that backward planning to avoid food intake characterizes binging in anorexia nervosa patients and facilitates binge craving by focusing attention on food [8]. Improving backward planning to avoid binge food intake might therefore explain the transition from ANR to ANB. At the end of each binge, ANB patients experience the refractory period that follows such food intake [11]. After several binges, failing the initial objective of avoiding binge food intake through improved planning might bring patients to focus on the optimization of the refractory period, and thus shifting from ANB to BN. The shift from BN to ANB could be similarly explained by a perceived non-satisfactory refractory period that would result in a strict avoidance of binge food intake. Second, assessment of these two strategies might facilitate differential diagnosis between BN and ANB when patients have a BMI close to the anorexia nervosa threshold. Finally, reorienting or disrupting planning during setting of binges and binge food intake may be therapeutic. To date, cognitive behavioral interventions that are the most effective treatments regarding binge eating [38] aim to prevent binge occurrence and likely miss mechanisms occurring during binges [39]. A behavioral intervention or the use of transcranial magnetic stimulation targeting brain areas associated with backward planning ability during binge food exposure might facilitate recovery.

### Limitations of the study

These results should be interpreted carefully, and further investigation is needed to understand the important role of planning and self control in binge eating.

First, patients were hospitalized and under medication. However medications were stable for more than one week, which do not affect performances at neuropsychological tasks [23], [24]. The possible selection bias due to the recruitment of inpatients is limited because over 70% of AN patients and 50% of BN patients experience at least one hospitalization and hospitalization is a recommended setting for cares in AN [40]–[43]. Finally, should be noticed that we reproduced several previously published results in these populations (supplementary results 3 in file S2).

We did not ask participants at the end of the study what they thought the purpose of the primes was. Participants may show different behavior in part due to demand effects, since they can easily tell that the experimenter is presenting them with different conditions. However, our results show that the effects of priming are different among groups, and (more importantly to exclude the demand effect) different within group of participants for different tasks.

Our tasks did not directly involve planning about food consumption, and thus the enhanced planning based on backward reasoning in BN and ANB patients in the binge food condition might have been only the consequence of an emotional reaction to binge food. We believe that this alternative interpretation is unlikely because BN, ANB and ANR patients share a common avoidance toward binge foods outside binges [1], and binge food cues do not lead systematically to binge eating in BN patients [1], [3].

Also, we did not assess the craving aroused by the cues nor patients' sensitivity to food rewards. Hence our data cannot rule out that enhanced planning ability based on backward reasoning observed in binging patients in the food condition, may result from a higher cognitive flexibility elicited by a stronger positive mood, due to craving and/or reward value of foods in these patients [44]–[46]. However, this hypothesis is unlikely because, first, binge foods are associated with a strong perceived threat [1], [3] in binging patients (as suggested by the high level of anxiety associated with food images in our experiment) that leaves no place for a strong positive mood; second, planning based on backward reasoning differs from other kinds of planning, such as forward planning, by the fact that it determines an optimal sequence of actions reasoning back from the end (unique and fixed) goal [22], [47]. Backward reasoning thus limits the use of exploration and flexible behavior [22], [47]. Accordingly patients' behavior toward foods and recruitment of cognitive skills are driven rather by motivation to binge than by reward value given to food [48].

## Supporting Information

File S1 **Provides additional details on task design, fitting methods and robustness analyses conducted.**
(DOC)

File S2 **Provides additional results, tables and figures.**
(DOC)
