# Supplementary material for: Improved Planning Abilities in Binge Eating
Source: PLoS One. 2014 Aug 22;9(8):e105657. doi: 10.1371/journal.pone.0105657 (PMC4141805; doi:10.1371/journal.pone.0105657)
Supplement: File S1 — Provides additional details on task design, fitting methods and robustness analyses conducted. (DOC) [file pone.0105657.s001.doc]

**Supplementary methods**

**Trial design for intertemporal discounting, Simon and Go/No-go tasks**

All trials of these three tasks shared a preconditioning part composed of a 500 ms fixation cross followed by a one second neutral/relaxing, binge food or fearful image followed by a second 500 ms fixation cross as for the Race game (figure 1A).

For the intertemporal discounting task, the preconditioning part was followed by two text options displayed simultaneously on the left and the right sides of the screen. One option referred to the immediate reward and the second one to the delayed one. Participants had to make their choice in 4 seconds maximum. Then a feedback with the participants' outcome was displayed for two seconds.

For the Simon task, the preconditioning part was followed by a horizontal arrow pointing toward the left or the right displayed on the screen (either left side or right side). Participants had to indicate toward which side of the screen the arrow was pointing. Participants had at most two seconds to provide their response once the arrow was displayed. If participants were too slow to answer, a feedback was displayed for two seconds. 50% of horizontal arrows were pointing toward the left and 50% of trials were incongruent. 120 trials were performed.

For the Go/No-go task, after the preconditioning part, a letter (V or M) was displayed at the center of the screen. Participants were instructed to respond as fast and as accurately as possible when a “V” was displayed (go trials) and to refrain from pressing the response button when a “M” was displayed (no-go trials). Participants had at most 4.5 seconds to provide their response. This was followed by a feedback (correct/incorrect) lasting two seconds. 90 out of the 120 trials were go trials.

**Fitting method for intertemporal discounting task**

For each choice, the probability of choosing an option, delayed or immediate, was modeled as a logistic function. Only options with immediate payoffs ranging between 1 and 9€ were included in the analysis. Because the traditional hyperbolic function (Equation s1.1) models participants' behavior when they switch preferences from delayed to immediate option , the power of the exponential of the logistic function at the switch (i.e. proba(choice=delayed option)=proba(choice=immediate option)=0.5) should be equal to the hyperbolic discounting function. By rewriting equation s1.1 so that f(k)=0, we obtained the probability functions of choosing the delayed and immediate options respectively (Equation s1.2, s1.3):

Immediate payoff= delayed payoff / (1+ k x delay) (Equation s1.1)

Proba (choice = delayed option) = e^a^ / (1 + e^a^) (Equation s1.2)

Proba (choice = immediate option) = 1 / (1 + e^a^) (Equation s1.2)

With a = immediate payoff - delayed payoff + k x immediate payoff x delay

For each participant, parameters were estimated by minimizing the error between fit and participants' choice with matlab fminsearch function. To avoid local minimum, we run the fminsearch function 5 times for each participant with 5 different initializations for k: 0.01, 0.1, 0.5, 1 and 2. The fit with the minimal error was selected. If participants always chose the same option in a condition, k was set to 0 for the absence of discount of the delayed option. Inversely, if participants chose only the immediate option in a condition, k was set to infinity for the discount of all delayed options.

**Reliability of SEM results: analysis of pattern of correlations**

The following associations were assessed with the Spearman correlation coefficient for each group (ANR, ANB and BN):

- Rate of success at the Race Game: between the food condition and the neutral or stress conditions
- "Food-specific" success rate in the Race game with "food-specific" reaction time at correct go trials or with "food-specific" anxiety arousal. Similar associations were assessed with food-stress differential instead of food-neutral differential.
- "Food-specific" reaction time at correct go trials with "food-specific" anxiety arousal. Similar association was assessed with food-stress differential instead of food-neutral differential.
- Coefficient of variation of reaction time at correct go trials between the food condition and the neutral or stress conditions.
- Reaction time at correct go trials between the food condition and the neutral or stress conditions.

We assessed the association of correlation coefficients between ANB and ANR groups, and between ANB and BN groups with a Spearman correlation coefficient. We compared the two correlation coefficients with the Fischer Z statistic.

To test the robustness of results (i.e. ANB are expected to be closer to ANR than to BN) we ran again this procedure removing the associations not directly related to planning, namely those that did not include the success rate at the Race game.
