# Supplementary material for: Improved Planning Abilities in Binge Eating
Source: PLoS One. 2014 Aug 22;9(8):e105657. doi: 10.1371/journal.pone.0105657 (PMC4141805; doi:10.1371/journal.pone.0105657)
Supplement: File S2 — Provides additional results, tables and figures. (DOC) [file pone.0105657.s002.doc]

**Supplementary results**

**Supplementary result 1: fitting appraisal**

*Convergence and goodness of fit of SEM analysis*

SEM provided a good fit for BN patients (p_chi2_=0.38; RMSEA=0.055). In ANB patients, the poor RMSEA (p_chi2_=0.13; RMSEA=0.12) could be due to the small sample size and did not lead to rejection of the model . Adding paths between food anxiety arousal and approach stability, and between objective anxiety and avoidance stability, provided a poorer fit for BN (p_chi2_=0.32; RMSEA=0.075) but a relatively better one for ANB group (p_chi2_=0.17; RMSEA=0.11). These additional paths gave similar results for the ANB group. Backward reasoning in the binge food condition at the Race game was associated with avoidance behavior (p=0.04), not with approach behavior (p=0.37).

*Robustness of patterns of correlations*

ANB pattern of correlations (supplementary methods §3 analysis of pattern of correlations) was substantially closer to the ANR one than to the BN one even after removing:

- correlations between coefficients of variation of reaction time at correct go trials in the different conditions (rho Spearman : r_ANB-ANR_=0.88, p=0.002 versus r_ANB-BN_=0.30, p=0.41),
- or correlations between reaction time at correct go trials in the different conditions (r_ANB-ANR_=0.81, p=0.008 versus r_ANB-BN_ =-0.41, p=0.25),
- or correlations between reaction time at correct go trials and anxiety arousal (r_ANB-ANR_ =0.90, p=0.0009 versus r_ANB-BN_ =0.19, p=0.61)
- or all these correlations from the pattern (r_ANB-ANR_ =0.89, p=0.03 versus r_ANB-BN_ =-0.60, p=0.24).

**Supplementary result 2 and interpretation: approach/avoidance behavior, the go/No-go task**

We investigated whether groups used distinct processes to control the repetition of a dominant behavior in food and neutral conditions. In that aim, we compared reaction times profile (mean and standard deviation (SD), a proxy of stability) at the Go/No-go task between food and neutral conditions. Mean reaction times at correct go trials in binge food and neutral conditions were highly correlated within the four groups: healthy controls: r=0.95, p<0.0001; BN: r=0.83, p<0.00001; ANB: r=0.74, p=0.0004; ANR: r=0.89, p=0.00001. In contrast, reaction time SDs at correct go trials in neutral and binge food conditions were highly correlated in the healthy group (r=0.81, p<0.0001), tended to be correlated in ANR (r=0.42, p=0.07) and ANB: r=0.39, p=0.05) groups and were not correlated in the BN group (r=-0.08, p=0.77). Association of reaction time stability between neutral and binge food conditions was significantly stronger for healthy controls than for AN patients (i.e. ANR+ANB) (r=0.81 versus r=0.43, p=0.03), the latter tending to be significantly stronger than for BN patients (r=0.43 versus r=-0.08, p=0.09). These results suggest that healthy controls engage the same process to repeat a dominant behavior in neutral and binge food conditions, BN patients engage distinct processes and ANB and ANR are in the middle: they engage partially the same processes.

**Supplementary result 3: comparison with literature**

In line with literature, we observed that non-binging participants (ANR and healthy participants) succeeded less often in stressful conditions than in neutral ones (figure 1C) , -and improved planning abilities (measured by the success rate at the Race game) were not associated with general food deprivation (measured by the time elapsed since the last meal) in BN (r=-0.24, p=0.33) and ANB patients also (r=-0.10, p=0.69, neither with BMI in ANB patients(r=-0.15, p=0.53). Moreover, ANR and ANB patients had similar success rates at the Race game in neutral condition (p=0.25, figure 1C). This result is in line with the one obtained with the tower of Hanoi task . No comparison with literature can be drawn for BN patients because previous studies used the Tower of London task that does not recruit backward reasoning ability .

That BN patients improve planning based on backward reasoning to promote large delayed rewards confirms the role of planning based on backward reasoning in the choice of large delayed rewards previously reported for the Race game in healthy participants .

We also observed the interference effect in neutral condition in the Simon task for BN patients and for healthy control women (difference of reaction time between correct incongruent and correct congruent trials, mean (SD): 63 (77) ms, p<0.004 and 60 (60) ms, p<0.0006 respectively, table 1).


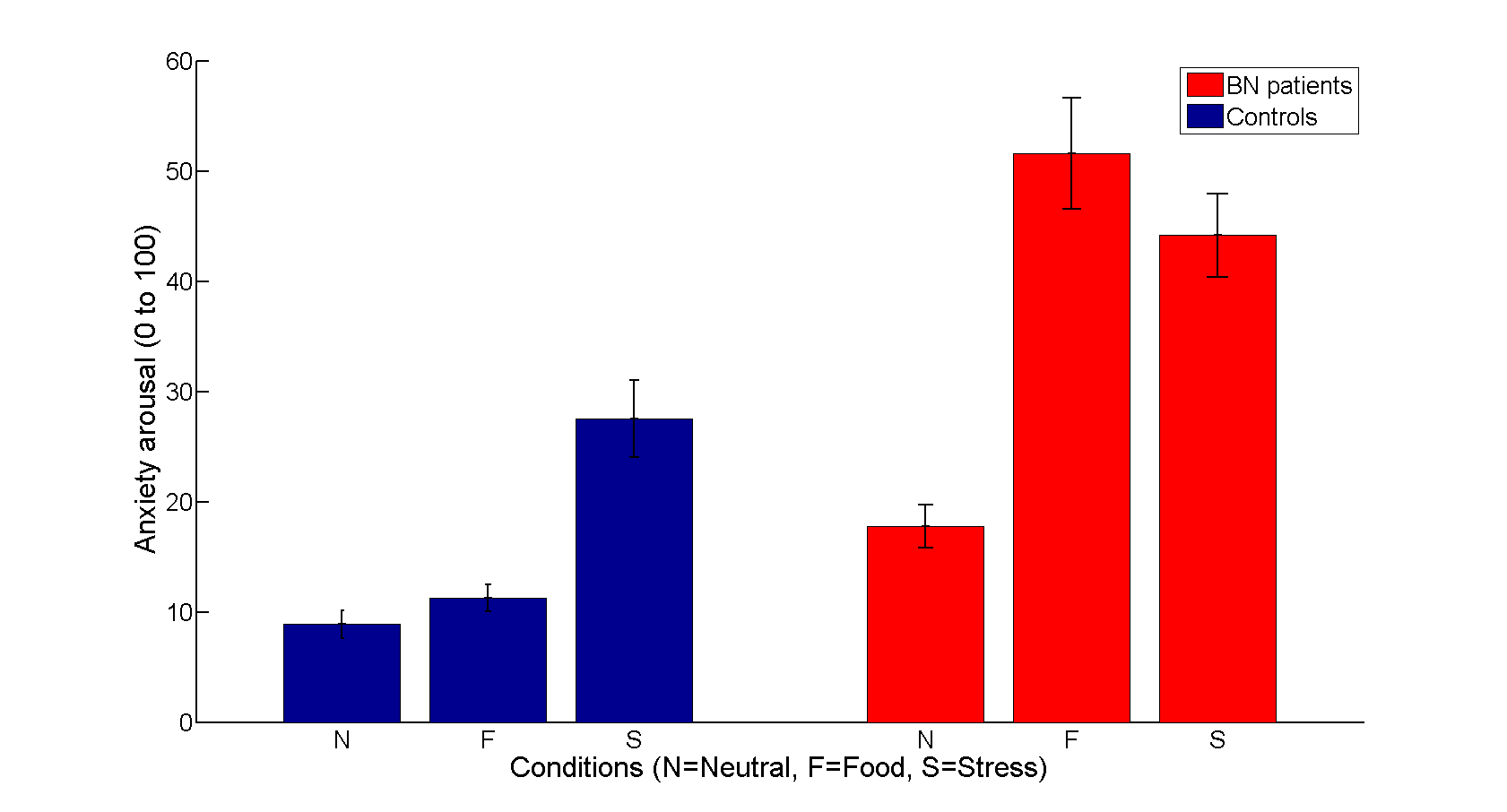

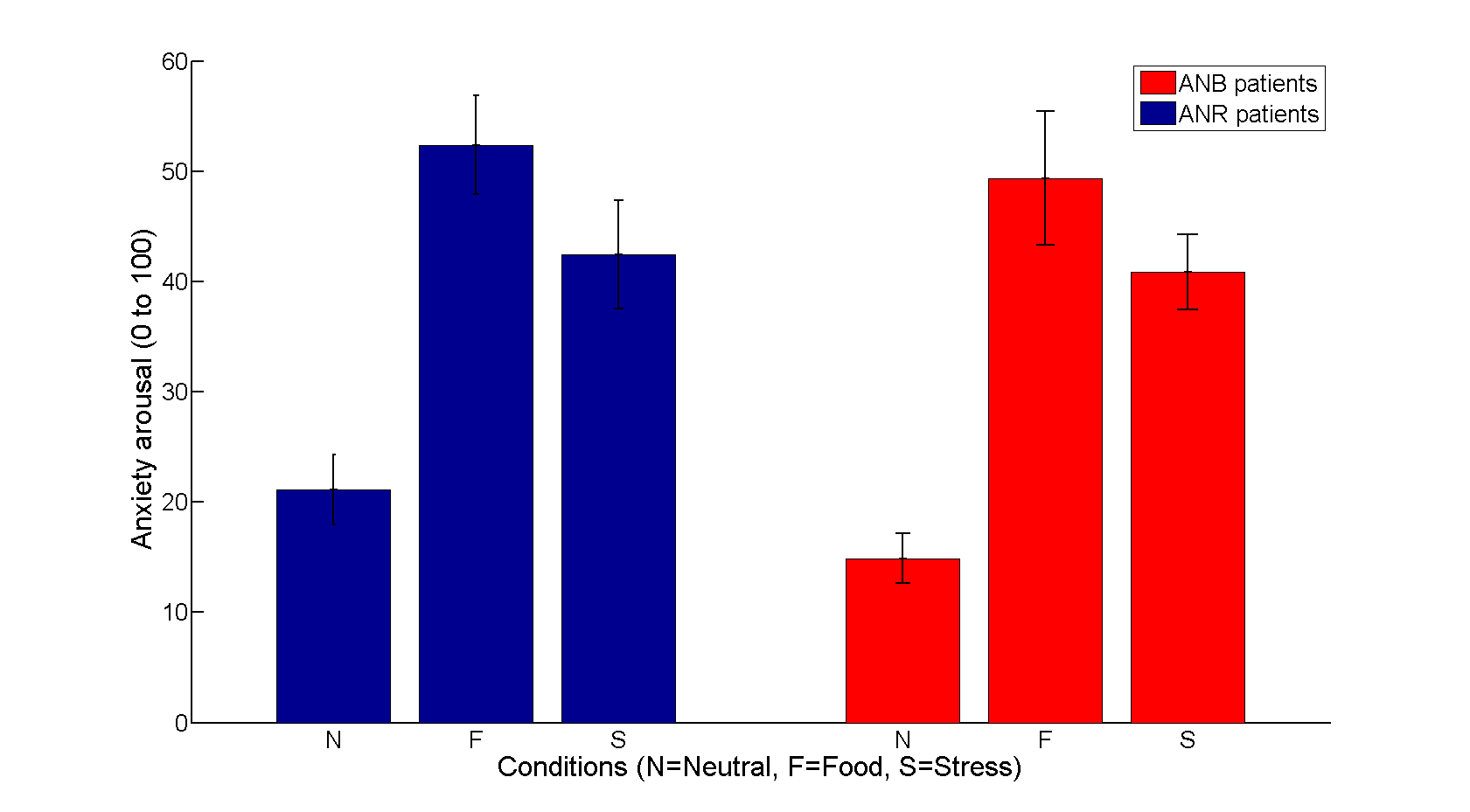


p=0.01

p=0.001

p=0.0001

p=0.0003

p=0.06

p=0.0002

p=0.02

p=0.0003

p=0.0002

p=0.0002


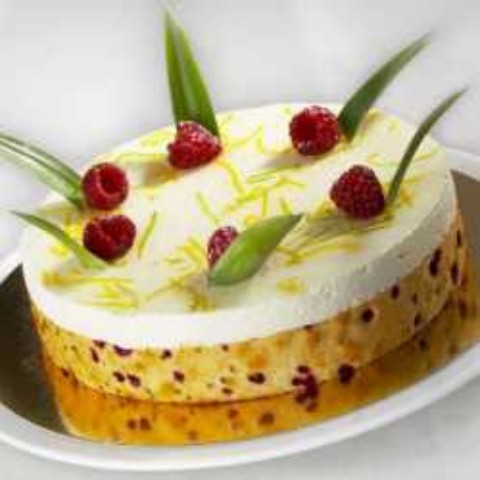

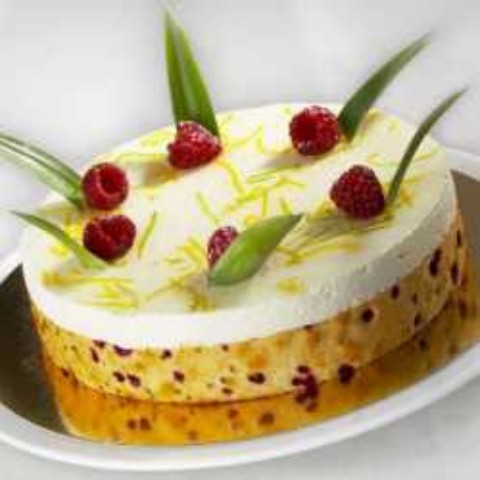

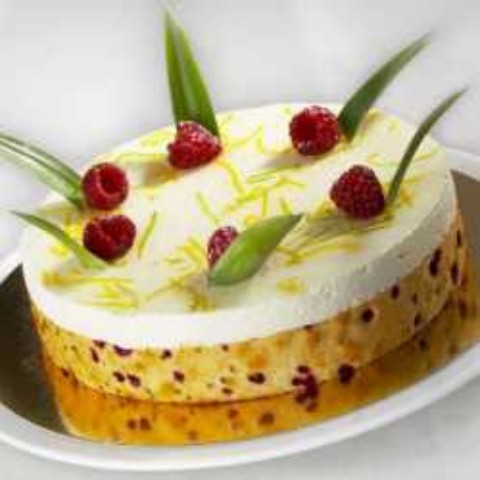

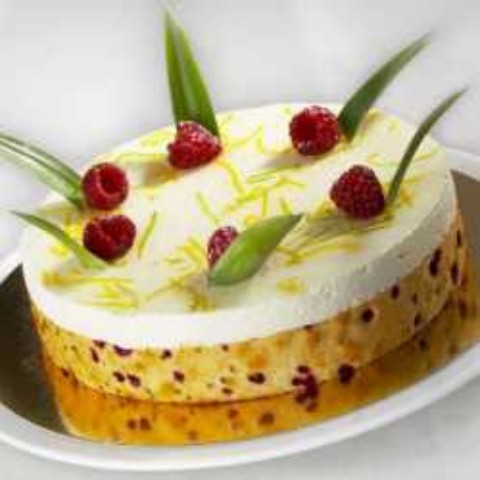

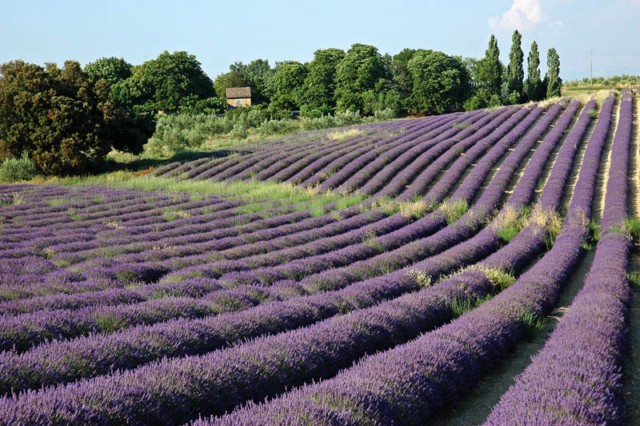

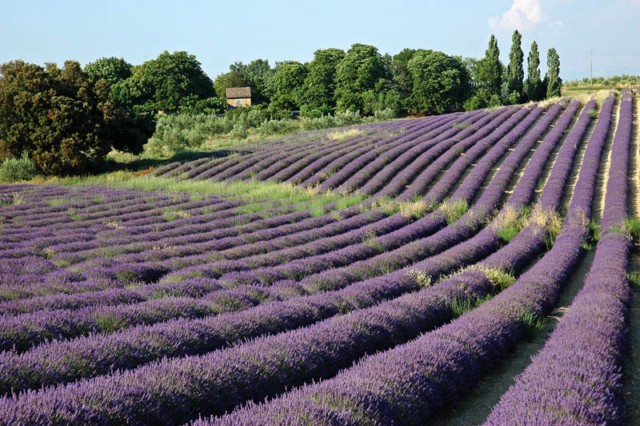

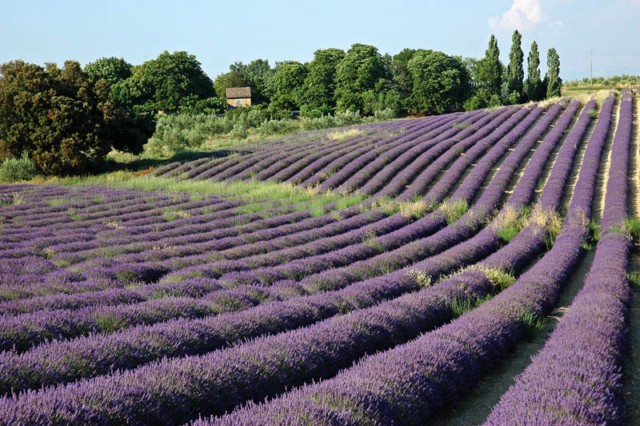

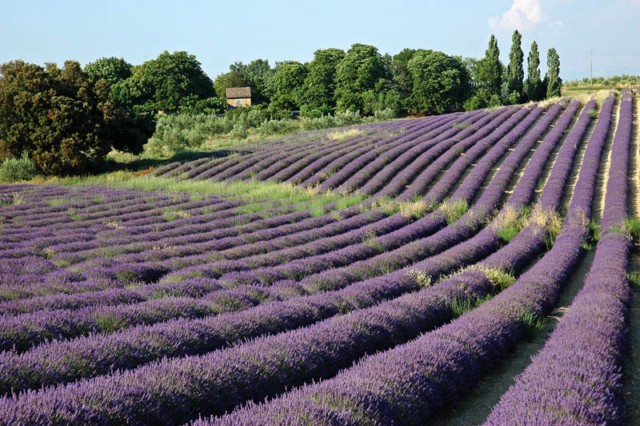

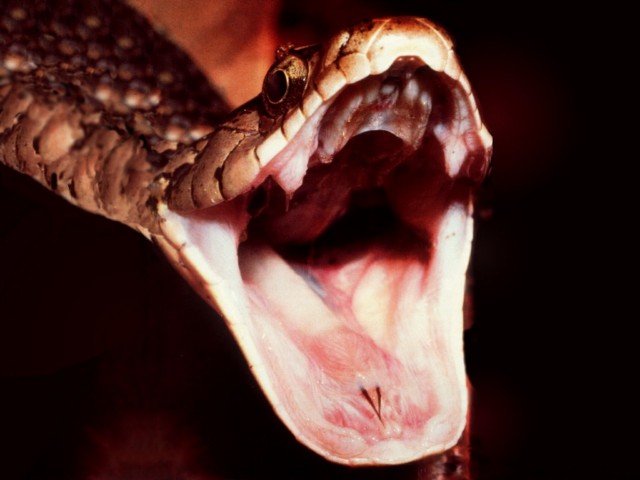

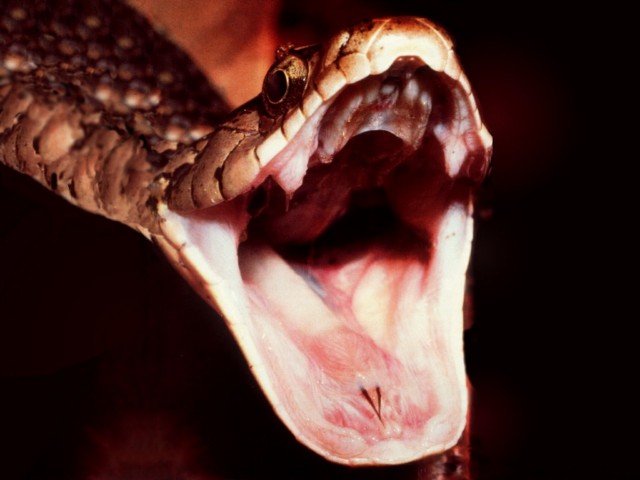

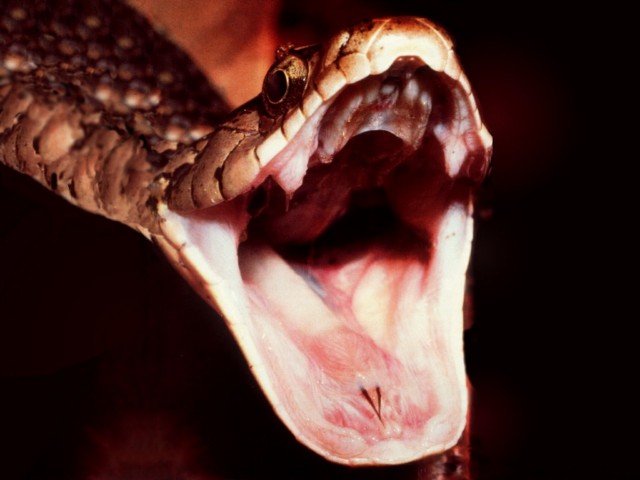

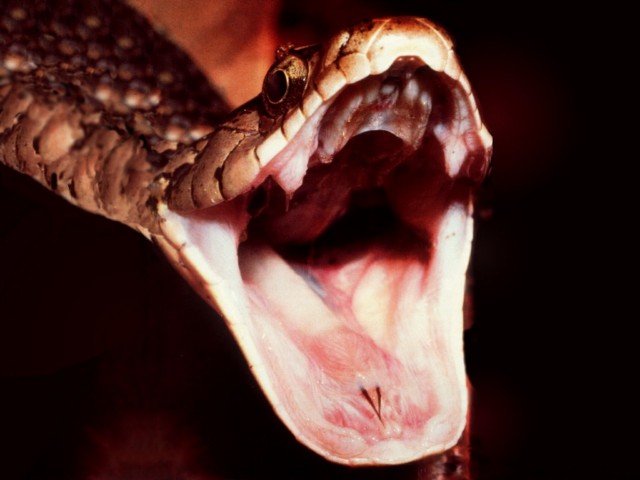


| Anxiety arousal (mean (SD)) | Bulimia nervosa  (n=18) | Controls (n=18) | p-value | Anorexia nervosa binging subtype (n=19) | Anorexia nervosa restrictive subtype (n=16) | p-value |
| --- | --- | --- | --- | --- | --- | --- |
| Neutral condition | 17.8 (8.3) | 8.9 (5.3) | 0.0008 | 14.9 (9.9) | 21.1 (12.7) | 0.17 |
| Food condition | 51.6 (21.5) | 11.3 (5.2) | < 0.0001 | 49.4 (26.3) | 52.4 (17.8) | 0.99 |
| Stress condition | 44.1 (16.1) | 27.5 (14.8) | 0.0034 | 40.8 (14.9) | 42.4 (19.5) | 1 |
| Difference between conditions  Food versus neutral | 33.8 (19.6) | 2.4 (3.5) | < 0.0001 | 34.5 (23.9) | 31.3 (26.1) | 0.63 |
| Stress versus neutral | 26.4 (15.0) | 18.6 (12.0) | 0.064 | 26.0 (12.5) | 21.3 (26.6) | 0.56 |
| Food versus stress | 7.4 (18.9) | -16.3 (12.3) | 0.0002 | 8.5 (20.2) | 9.9 (24.9) | 0.99 |

Supplementary figure SF1: anxiety arousal rated from 0 (no anxiety) to 100 (highest anxiety ever felt) generated by images displayed during the Race game (mean and standard error of the mean for the graphic) in bulimia nervosa (BN), anorexia nervosa binging subtype (ANB) and anorexia nervosa restrictive subtype (ANR) patients and healthy controls. Only corrected p-values < 0.1 are reported on the graphic. All p-values are corrected for multiple testing (Benjamini-Hochberg correction).


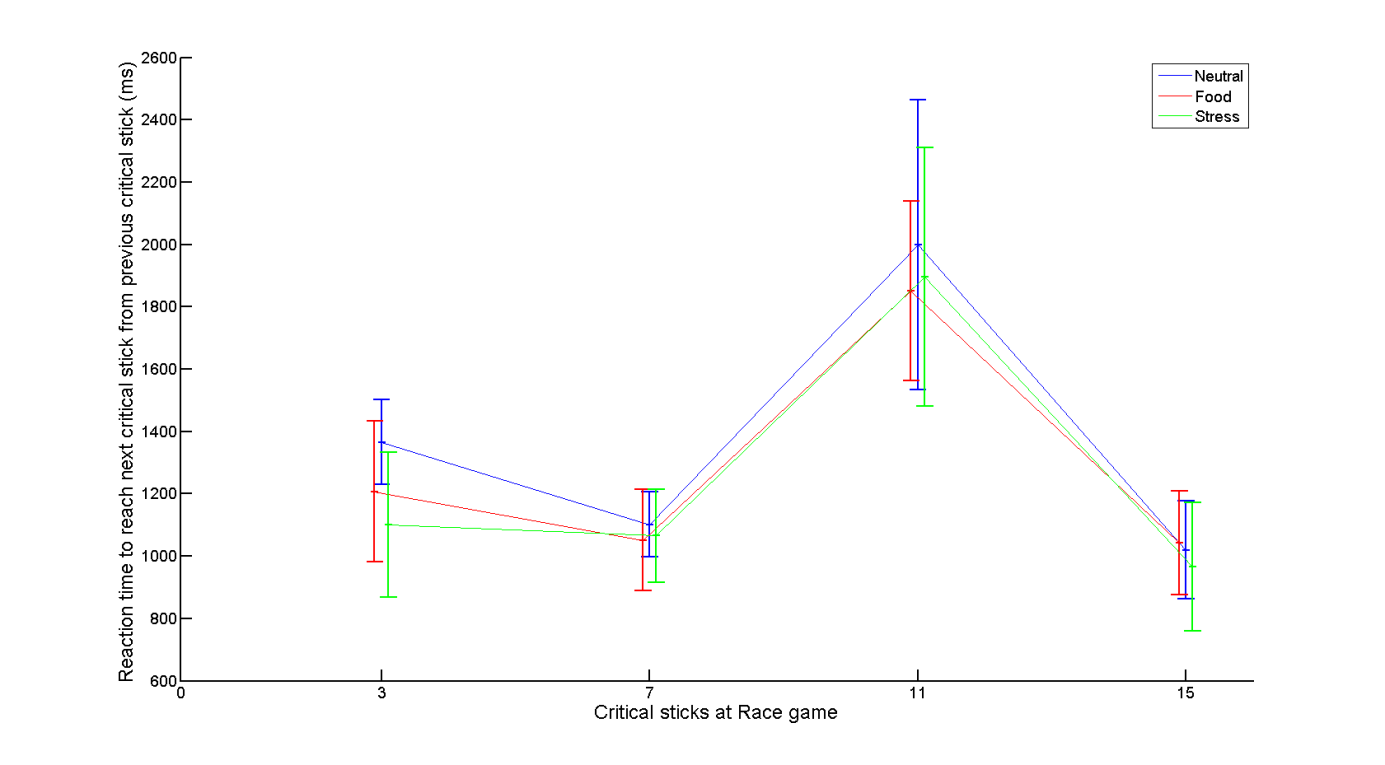


p=0.006

p=0.09

p=0.006

p=0.05

p=0.006

p=0.05

p=0.06

p=0.07

p=0.07

2A: anorexia nervosa restrictive subtype patients (n=16)


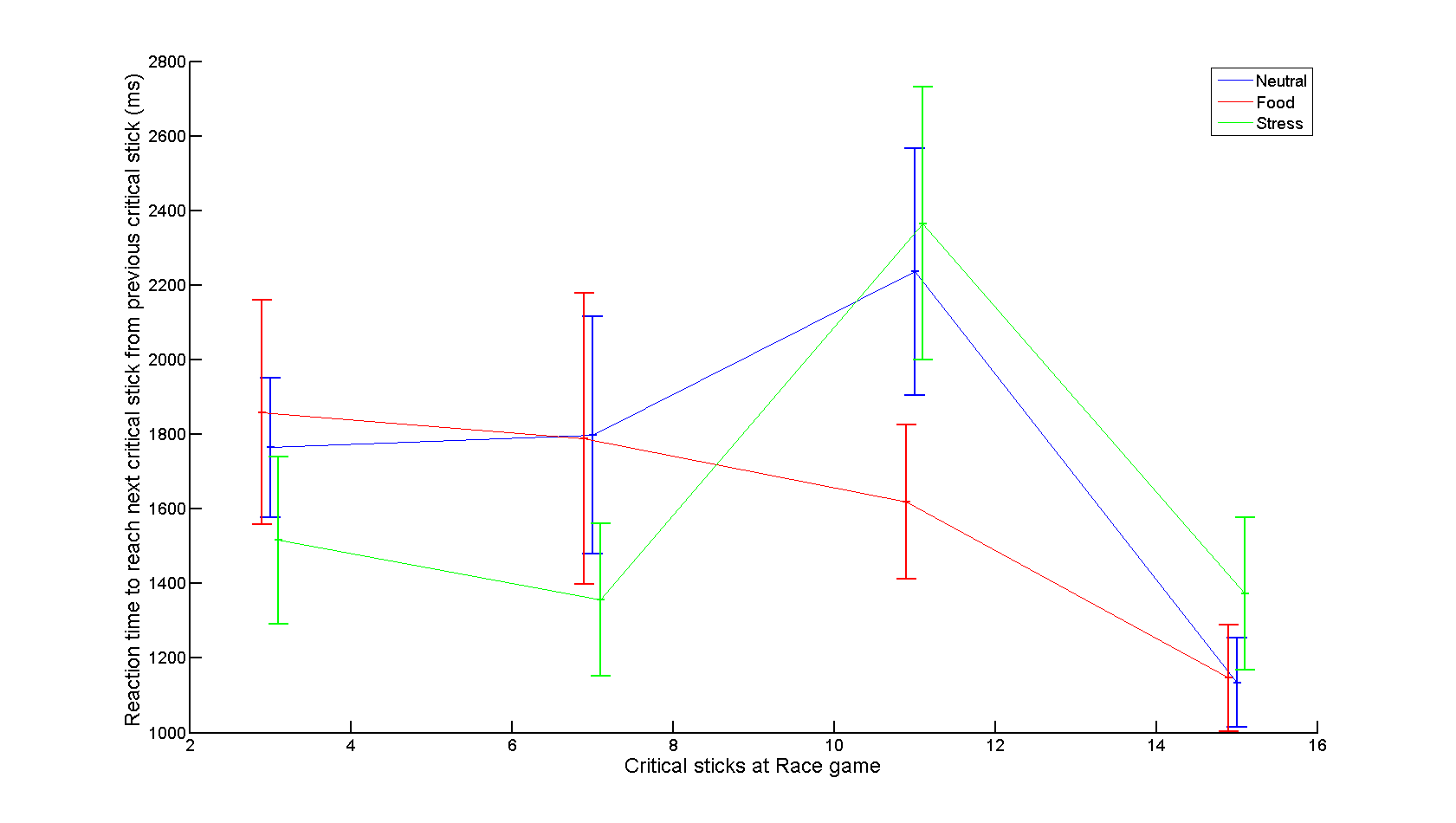


p=0.008

p=0.04

p=0.06

p=0.02

p=0.06

2B: anorexia nervosa binging subtype patients (n=19)


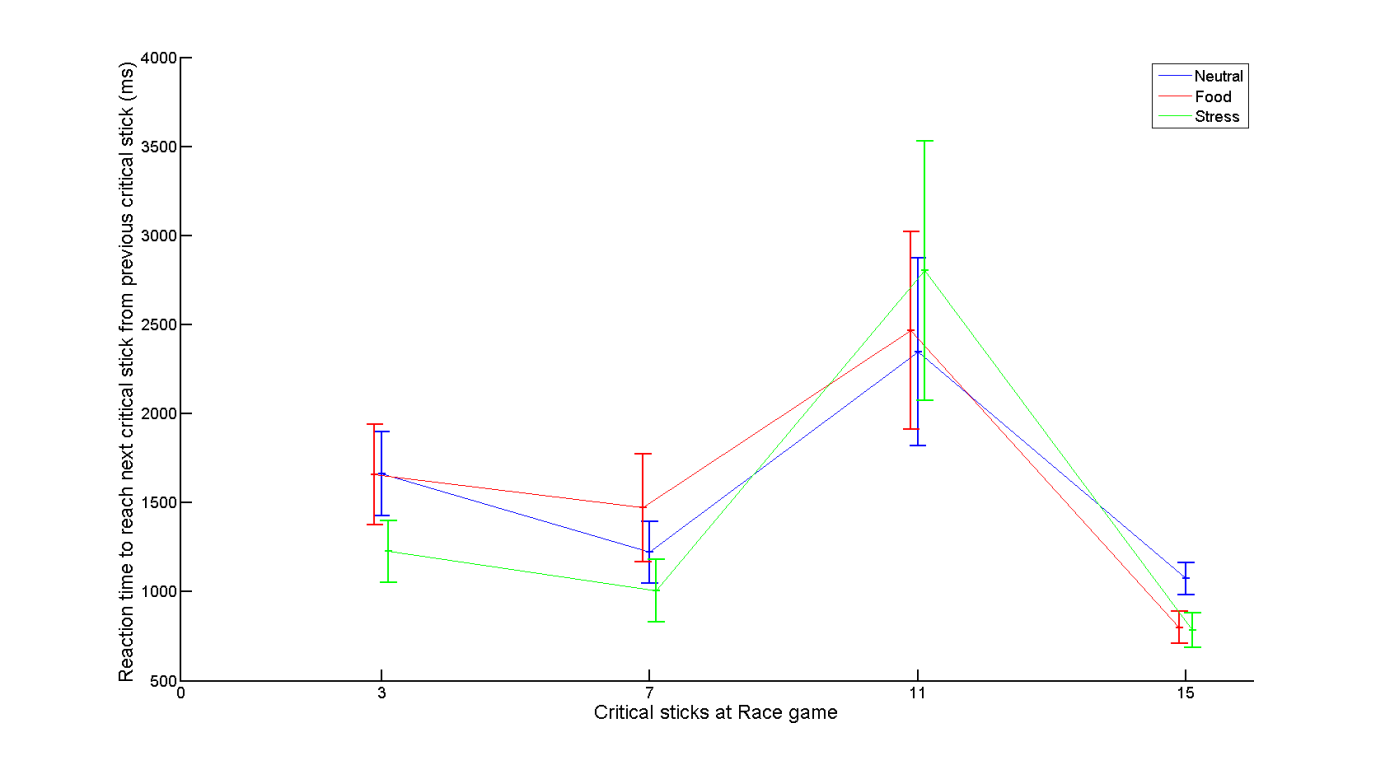


p=0.001

p=0.09

p=0.01

p=0.02

p=0.001

p=0.001

p=0.001

p=0.001

2C: bulimia nervosa patients (n=18)


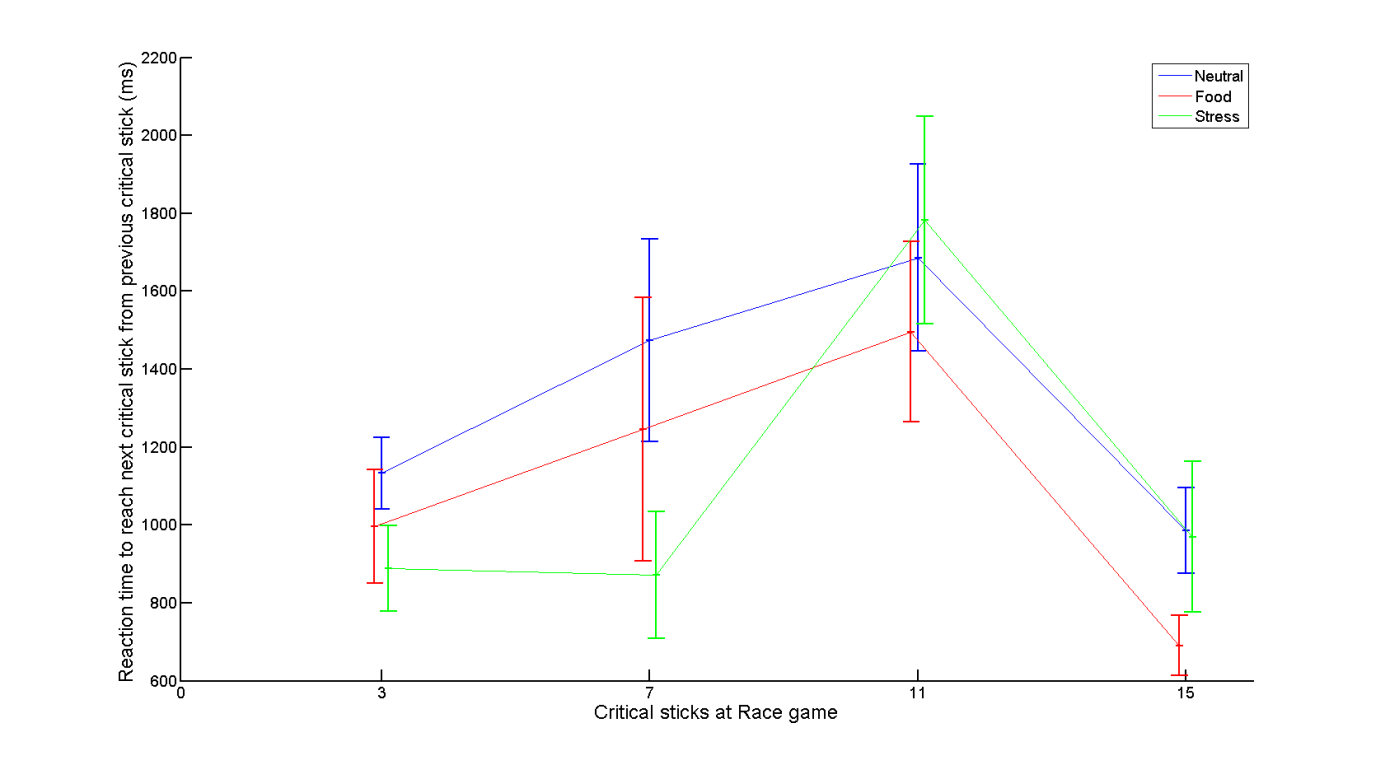


p=0.03

p=0.005

p=0.05

p=0.005

p=0.005

p=0.005

p=0.008

2D: healthy controls (n=18)

Supplementary figure SF2: reaction time at the Race game between two consecutive critical sticks (1 to 3, 4 to 7, 8 to 11 and 12 to 15) in each condition (neutral in blue, binge food in red and stress in green) for each group (2A: anorexia nervosa restrictive subtype, 2B: anorexia nervosa binging subtype, 2C: bulimia nervosa, 2D: healthy controls). p-values corrected for multiple testing (Benjamini-Hochberg correction). p values < 0.10 are reported.

| **ST1A:** Socio-demographic characteristics | n | Bulimia nervosa | n | Controls | n | Anorexia nervosa binging subtype | n | Anorexia nervosa restrictive subtype | p-value |
| --- | --- | --- | --- | --- | --- | --- | --- | --- | --- |
| Brothers, nb | 18 | 1 (0.82) | 18 | 1.28 (0.99) | 19 | 0.632 (0.666) | 16 | 0.69 (0.68) | 0.12 |
| Sisters, nb | 18 | 0.67 (0.94) | 18 | 0.67 (0.88) | 19 | 0.789 (0.893) | 16 | 0.75 (0.83) | 0.94 |
| Living status, n (%) | 17 |  | 17 |  | 17 |  | 15 |  | 0.11 |
| Alone |  | 7 (41.2) |  | 2 (11.8) |  | 4 (23.5) |  | 3 (20) |  |
| With family |  | 7 (41.2) |  | 6 (35.3) |  | 6 (35.3) |  | 9 (60) |  |
| Cohabitation |  | 2 (11.8) |  | 7 (41.2) |  | 6 (35.3) |  | 1 (6.67) |  |
| With a roommate/Other |  | 1 (5.88) |  | 2 (11.8) |  | 1 (5.88) |  | 2 (13.3) |  |
| Parental marital status (divorce), n (%) | 16 | 6 (37.5) | 16 | 5 (31.3) | 17 | 5 (29.4) | 15 | 6 (40) | 0.92 |
| Maternal socio-professional status, n(%) | 14 |  | 18 |  | 14 |  | 13 |  | 0.27 |
| INSEE 1 or 2 |  | 0 (0) |  | 1 (5.56) |  | 1 (7.14) |  | 1 (7.69) |  |
| INSEE 3 |  | 1 (7.14) |  | 4 (22.2) |  | 3 (21.4) |  | 1 (7.69) |  |
| INSEE 4, 5 or 6 |  | 7 (50) |  | 11 (61.1) |  | 4 (28.6) |  | 10 (76.9) |  |
| INSEE 7 or 8 |  | 6 (42.9) |  | 2 (11.1) |  | 6 (42.9) |  | 1 (7.69) |  |
| Source of financial income | 16 |  | 17 |  | 14 |  | 15 |  | 0.33 |
| With own income, n(%) |  | 9 (56.3) |  | 8 (47.1) |  | 7 (50) |  | 3 (20) |  |
| With parental financial support, n(%) |  | 6 (37.5) |  | 7 (41.2) |  | 6 (42.9) |  | 11 (73.3) |  |
| With fellowship, n(%) |  | 0 (0) |  | 2 (11.8) |  | 0 (0) |  | 1 (6.67) |  |
| Other, n(%) |  | 1 (6.25) |  | 0 (0) |  | 1 (7.14) |  | 0 (0) |  |

| **ST1B:** Physiological characteristics | Bulimia nervosa  (n=18) | Controls (n=18) | p-value | Anorexia nervosa binging subtype (n=19) | Anorexia nervosa restrictive subtype (n=16) | p-value |
| --- | --- | --- | --- | --- | --- | --- |
| Duration between last meal and Race game assessment , mn | 103 (37.4) | 70.5 (37.4) | 0.45 | 103 (37.4) | 104 (37.4) | 0.74 |
| Body Mass Index (kg/m^2^) | 20.6 (3.19) | 21.5 (3) | 0.19 | 17.6 (1.84) | 15.3 (1.19) | 0.0003 |

Supplementary table ST1: socio demographic (ST1A) and physiological characteristics (ST1B) of the four groups. Mean (standard deviation) are reported for quantitative parameters.

INSEE #: socio-professional category according to the "Institut National de la Statistique et des Etudes Economiques": 1,2: Farmer, craftsman, shopkeeper and large retailer, chairman and managing director; 3:senior executive, manager; 4,5,6: intermediate jobs, employees and workers; 7,8: retired, without any job.

| Difference between conditions, % | Bulimia nervosa (n=18) | Controls (n=18) | p-value | Anorexia nervosa binging subtype (n=19) | Anorexia nervosa restrictive subtype (n=16) | p-value |
| --- | --- | --- | --- | --- | --- | --- |
| food versus neutral | 15 (15.4) | 1.1 (21.7) | 0.035 | 6.5 (20.1) | -2.6 (30.7) | 0.082 |
| stress versus neutral | 1.7 (23.3) | -10 (27.2) | 0.23 | -2.5 (24.7) | -14 (21.2) | 0.058 |
| food versus stress | 13 (21.7) | 11 (23.0) | 0.88 | 9.0 (27.1) | 12 (28.5) | 0.68 |

Supplementary table ST2: Differences in success rate at the Race game between conditions for binging patients (bulimia nervosa and anorexia nervosa binging subtype) compared with their respective controls (healthy controls, anorexia nervosa restrictive subtype). Mean (standard deviation) are reported.

|  | Bulimia nervosa (n=18) | Controls (n=18) | Anorexia nervosa binging subtype (n=19) | Anorexia nervosa restrictive subtype (n=16) |
| --- | --- | --- | --- | --- |
| Neutral condition | 0.66 ** | 0.74 *** | 0.84 *** | 0.71** |
| Food condition | 0.49 * | 0.51 * | 0.91 *** | 0.82 *** |
| Stress condition | 0.65 ** | 0.83 *** | 0.88 *** | 0.65 ** |

Supplementary table ST3: Association between success rate and backward reasoning at the Race game in the four groups of participants and in each condition. Spearman correlation coefficients (p-value corrected for multiple testing (Benjamini-Hochberg correction) are reported. * p value < 0.05; ** p value < 0.01; *** p value < 0.001

| Starting point of the arrow | End point of the arrow | BN coefficient | ANB coefficient |
| --- | --- | --- | --- |
| Attractive behavior | RT F | 0.98** | 0.89** |
| Avoidance behavior | RT F | 0.16 | 0.41 |
| Attractive stability | CV F | 0.98** | 0.98** |
| Avoidance stability | CV F | 0.22 | 0.22 |
| Objective anxiety | Anxiety arousal F | 1** | 1** |
| Attractive behavior | Attractive stability | 0.59** | 0.75* |
| Avoidance behavior | Avoidance stability | -0.99** | -0.98** |
| Anxiety arousal F | Attractive behavior | -0.1 | 0.01 |
| Objective anxiety | Avoidance behavior | -0.38 | 0.18 |

Supplementary table ST4: standardized coefficients of the paths of model of figure 3A and not reported in figure 3B for Bulimia Nervosa (BN) and Anorexia Nervosa Binging subtype (ANB) patients.

**: p<0.05; *: p<0.1

**References**

1. Green L, Myerson J (1996) Exponential Versus Hyperbolic Discounting of Delayed Outcomes: Risk and Waiting Time. American Zoologist 36: 496-505.

2. Savalei V, Bentler P (2006) Structural Equation Modeling. In: R G, M V, editors. The Handbook of Marketing Research Sage.

3. Taylor SE, Pham LB, Rivkin ID, Armor DA (1998) Harnessing the imagination. Mental simulation, self-regulation, and coping. Am Psychol 53: 429-439.

4. Fowler L, Blackwell A, Jaffa A, Palmer R, Robbins TW, et al. (2006) Profile of neurocognitive impairments associated with female in-patients with anorexia nervosa. Psychol Med 36: 517-527.

5. Galimberti E, Fadda E, Cavallini MC, Martoni RM, Erzegovesi S, et al. (2013) Executive functioning in anorexia nervosa patients and their unaffected relatives. Psychiatry Res 208: 238-244.

6. Alvarado-Sanchez N, Silva-Gutierrez C, Salvador-Cruz J (2009) Visoconstructive deficits and risk of developing eating disorders. Span J Psychol 12: 677-685.

7. Brand M, Franke-Sievert C, Jacoby GE, Markowitsch HJ, Tuschen-Caffier B (2007) Neuropsychological correlates of decision making in patients with bulimia nervosa. Neuropsychology 21: 742-750.

8. Goel V, Grafman J (1995) Are the frontal lobes implicated in "planning" functions? Interpreting data from the Tower of Hanoi. Neuropsychologia 33: 623-642.

9. Burks SV, Carpenter JP, Goette L, Rustichini A (2009) Cognitive skills affect economic preferences, strategic behavior, and job attachment. Proc Natl Acad Sci U S A 106: 7745-7750.

10. Frederick S (2005) Cognitive reflection and decision making. Journal of economic perspectives 19: 25-42.

11. Marsh R, Steinglass JE, Gerber AJ, Graziano O'Leary K, Wang Z, et al. (2009) Deficient activity in the neural systems that mediate self-regulatory control in bulimia nervosa. Arch Gen Psychiatry 66: 51-63.
